# Revealing the Differences in Taste/Mouthfeel Quality of Raw Pu-erh Tea Under Different Drying Methods Based on Nontargeted and Targeted Metabolomics

**DOI:** 10.3390/foods15111918

**Published:** 2026-05-28

**Authors:** Zhengfei Luo, Linlong Ma, Faxing Wan, Xueyan Liu, Yangtao Zhang, Ting Xu, Qiqian Su, Dan Cao, Yanli Liu, Xiaomei Yan, Yanhong Liu, Xiaofang Jin

**Affiliations:** 1 Yunnan Key Laboratory of Tea Germplasm Conservation and Utilization in the Lancang River Basin, College of Biotechnology and Engineering, West Yunnan University, Lincang 677000, China; luophy@126.com (Z.L.); 18468167691@163.com (X.L.); qiqian_su@163.com (Q.S.); 2 Key Laboratory of Tea Resources Comprehensive Utilization, Ministry of Agriculture and Rural Affairs, Fruit and Tea Research Institute, Hubei Academy of Agricultural Sciences, Wuhan 430064, China; malinlong@hbaas.com (L.M.); wanfaxing@163.com (F.W.); skyiswide@163.com (D.C.); zkslyl@163.com (Y.L.); yanxiaomei0329@163.com (X.Y.); 3 College of Horticulture and Gardening, Yangtze University, Jinzhou 434025, China; 4 Lincang Inspection Testing and Certification Institute, Lincang 677000, China; 13988383009@163.com

**Keywords:** raw Pu-erh tea, drying methods, taste/mouthfeel quality, metabolomics

## Abstract

Drying plays a critical role in shaping tea quality. This study systematically investigated the differences in the taste/mouthfeel quality of hot-air drying raw Pu-erh tea (HDRPT), pan-fired drying raw Pu-erh tea (PDRPT), and sun drying raw Pu-erh tea (SDRPT). The results showed that SDRPT exhibited a mellow and thick mouthfeel with a sweet aftertaste, and significantly higher intensities of umami and sweetness compared to PDRPT and HDRPT. The relatively higher contents of amino acids such as theanine, glutamic acid, aspartic acid, and glutamine, along with water-soluble sugars, and relatively lower contents of catechins, including ECG and EGCG, were identified as key material bases contributing to the unique taste/mouthfeel quality of SDRPT. A total of 1987 non-volatile components were detected, from which 137 differential non-volatile components were screened. Among these, 75 components in SDRPT showed significant differences from both PDRPT and HDRPT, with the majority exhibiting higher relative contents in SDRRT, particularly notable among lipids, phenolic acids, and terpenoids. Furthermore, all 75 differential components were significantly correlated with at least one taste/mouthfeel attribute, with most showing significant positive correlations with umami, sweetness, and sourness, and significant negative correlations with bitterness and astringency. These findings provide a theoretical foundation for understanding the mechanism by which drying techniques influence the taste/mouthfeel quality formation of raw Pu-erh tea, and offer a scientific basis for improving its quality.

## 1. Introduction

Pu-erh tea is a renowned local tea unique to Yunnan Province, China. It is highly popular among consumers worldwide for its unique flavor qualities and remarkable health benefits [1,2]. Based on processing techniques and quality characteristics, Pu-erh tea is classified into raw Pu-erh tea and ripe Pu-erh tea [3]. Raw Pu-erh tea is produced from the fresh leaves of the *Camellia sinensis* var. assamica in Yunnan, processed through steps such as fixing, rolling, and drying. It can be consumed directly as a type of green tea or transformed into aged Pu-erh tea through natural fermentation, or into ripe Pu-erh tea through artificial fermentation [3,4]. Although the processing method of raw Pu-erh tea is similar to that of green tea, it exhibits distinct differences in liquor color, aroma, taste, and mouthfeel compared to green teas such as ‘Xihu Longjing’ and ‘Huangshan Maofeng’. Raw Pu-erh tea is characterized by a bright yellow liquor, a strong and mellow mouthfeel with a sweet aftertaste, and a lasting aroma [5].

Drying is the final step in tea processing and a crucial stage for the formation and stabilization of flavor components [6,7]. The purpose of drying is to remove moisture for easier storage and simultaneously enhance flavor quality through complex thermochemical reactions [7]. Different drying temperatures and methods alter the composition and proportion of active substances such as polyphenols, sugars, and amino acids in tea, thereby affecting the balance and characteristic profile of its taste/mouthfeel [8]. Ye et al. [9] found that sun drying has the effect of reducing the bitterness and astringency of crude Ancha tea, attributing this to a decrease in the contents of (-)-epicatechin gallate, (-)-epigallocatechin gallate, and kaempferol-3-rutinoside, as well as an increase in the contents of kaempferol-3-glucoside, quercetin-3-glucoside, and myricetin-3-galactoside. Lu et al. [10] discovered that Yunnan Congou black tea dried with hot air produced a tea infusion that was higher in brightness, redness, and sweetness, whereas sun-drying significantly increased the content of tea pigments and bitterness, and pan-firing resulted in a more astringent tea infusion. Tu et al. [11] indicated that hot air drying can reduce the contents of amino acids and flavonols in green tea but can increase the contents of flavonoid glycosides.

In the production of raw Pu-erh tea, three traditional drying methods are mainly used, including hot-air drying, pan-fired drying, and sun drying, among which sun drying is the most widely applied method [12,13]. Hot-air drying and pan-fired drying are carried out at relatively high temperatures (with pan-fired drying reaching a higher temperature), resulting in a short drying time for the tea leaves [10,11]. In contrast, sun drying is typically conducted under room-temperature and light-exposed conditions with a much longer drying time, leading to significant differences in the flavor quality of the tea products processed by these three methods [10,14]. Although all three methods are utilized in production, there is currently a lack of systematic research that conducts a parallel comparison of the three within the same experimental framework, and the understanding of how different drying methods affect the taste/mouthfeel quality of raw Pu-erh tea remains unclear. Therefore, this study used fresh leaves (one bud and two leaves) of the Mengku large-leaf tea variety (*C. sinensis* var. assamica) as materials. After spreading, fixation, and rolling, the leaves were processed into hot-air drying raw Pu-erh tea (HDRPT), pan-fired drying raw Pu-erh tea (PDRPT), and sun drying raw Pu-erh tea (SDRPT) using the three drying methods. By employing non-targeted metabolomics, targeted metabolomics, and sensory evaluation methods, the biochemical components of raw Pu-erh tea under different drying methods were systematically analyzed, and key differential biochemical components were identified. The findings will not only help elucidate the effects of different drying methods on the taste/mouthfeel quality of raw Pu-erh tea and reveal the “processing-component-sensory quality” correlation mechanism but also provide clear chemical indicators for the future directional regulation of drying processes.

## 2. Materials and Methods

### 2.1. Experimental Materials

In April 2023, fresh leaves of the Mengku large-leaf variety (one bud and two leaves) were picked at the experimental base of West Yunnan University (located in Mengku Town, Shuangjiang County). Next, the spreading process involved placing fresh leaves at a density of 0.75 kg/m^2^ under conditions of room temperature 18~20 °C, and approximately 75% relative humidity until the moisture content in the leaves decreased to around 65%. The spread leaves were processed in a drum fixation machine (6CST-80, Hangzhou Chunjiang Tea Machinery Co., Ltd., Zhejiang, China) at 280 °C for 2 min. Rolling was carried out using a rolling machine (6CR-65, Zhejiang Chunjiang Tea Machinery Co., Ltd., Hangzhou, China) for 30 min. For drying, PDRPT was processed in a drum fixation machine (6CST-80, Zhejiang Chunjiang Tea Machinery Co., Ltd., Hangzhou, China) at 180~250 °C for 25 min, followed by 90~95 °C for 30 min. HDRPT was dried in a hot-air dryer (6CHM-901, Zhejiang Fuyang Tea Machinery Co., Ltd., Hangzhou, China) at 110~120 °C for 10~15 min, followed by 80 °C for 15~20 min. SDRPT was dried in bamboo trays under sunlight at 25~30 °C for 4~6 h, with the leaves turned every hour. The moisture content of all tea samples was measured using a rapid moisture analyzer (HS153, Mettler Toledo, Zurich, Switzerland) and maintained below 6%. Finally, 500 g were randomly selected from each sample in triplicate and stored in a −80 °C freezer for further use.

### 2.2. Chemicals

The standards for epigallocatechin gallate (EGCG), epigallocatechin (EGC), catechin (C), epicatechin (EC), epicatechin-3-gallate (ECG), gallic acid (GA), catechin gallate (CG), gallocatechin (GC), gallocatechin gallate (GCG), caffeine, theanine, glutamic acid, and glutamine were purchased from Shanghai Yuanye Biotechnology Co., Ltd., Shanghai, China. The standards for amino acids (aspartic acid, serine, arginine, glycine, histidine, threonine, alanine, proline, valine, lysine, isoleucine, leucine, phenylalanine) were purchased from Waters (Milford, MA, USA). HPLC-grade methanol, acetonitrile, formic acid, and acetic acid were purchased from Thermo Fisher Scientific (Waltham, MA, USA). Ninhydrin, folin–phenol, glucose, anthrone, alum, sucrose, citric acid, and other chemical reagents were obtained from Shanghai Sinopharm Chemical Reagent Co., Ltd., Shanghai, China.

### 2.3. Experiment Methods

#### 2.3.1. Sensory Evaluation

A total of 9 trained personnel (23–48 years old, 5 males and 4 females) participated in our study, including 3 (2 males and 1 female) for traditional sensory evaluation and 8 (4 males and 4 females) for main taste/mouthfeel intensity analysis. All evaluators were in good health, non-smokers, and not currently taking any medication, with no other exclusion criteria applied. The taste/mouthfeel characteristics of the tea samples were evaluated using traditional sensory evaluation following GB/T 23776-2018 [15], with the sensory vocabulary based on GB/T 14487-2017 [16]. Briefly, 3 g of tea samples were brewed in 150 mL of boiling water for 5 min, after which the infusion was filtered, and its taste/mouthfeel was evaluated. Meanwhile, the intensities of four basic taste attributes (bitterness, umami, sweetness, and sourness) and one mouthfeel attribute (astringency) of the tea infusion were evaluated using the methodology described by Li et al. (2019) [17]. A total of 8 evaluators underwent three rounds of training on bitterness, astringency, umami, sweetness, and sourness to enhance their sensitivity to these intensities. A 10-point scale was used to rate the intensity of these five attributes in the tea infusion, where 0 = absent, 3 = weak, 5 = moderate, 7 = strong, and 10 = very strong. Caffeine, alum, glutamic acid, sucrose, and citric acid were used as standard reference substances for bitterness, astringency, umami, sweetness, and sourness, respectively. To maintain objectivity, all samples were anonymized with three-digit codes and randomly served for assessment, and each sample was evaluated in triplicate.

#### 2.3.2. Determination of Main Biochemical Components

The total free amino acid content was determined by the ninhydrin colorimetric method, with L-glutamic acid as the reference substance (GB/T 8314-2013) [18]. The content of tea polyphenols was determined by the Folin–phenol colorimetric method, with gallic acid as the reference substance (GB/T 8313-2018) [19]. The determination of water-extract content was carried out with reference to GB/T 8305-2013 [20]. The content of water-soluble sugar was determined according to the method of Li (2018), using glucose as the reference substance [21].

The determination method for catechin content was the same as that for caffeine content. The specific procedure was as follows: A mass of 0.05 g of the sample was weighed and transferred into a 10 mL centrifuge tube. A volume of 5 mL of a 70% methanol–water solution was added, then the tube was placed in a water bath at 70 °C for 20 min. After incubation, the mixture was centrifuged at 3500 rpm for 5 min, and the supernatant was collected in a 10 mL volumetric flask. The extraction was repeated once more with the same volume of 70% methanol–water solution to ensure complete extraction. The combined supernatants were brought to a final volume of 10 mL with a 70% methanol–water solution, followed by filtration through a 0.22 µm filter membrane for subsequent HPLC analysis. The chromatographic equipment and conditions were in accordance with those described in our previous study [22].

The Waters AccQ•Tag method and a Waters AccQ•Tag column (Nova-Pak C18, 4 µm, 150 mm × 3.9 mm, Waters, Milford, MA, USA) were used to detect various amino acids according to the protocol provided with the AccQ•Fluor Reagent Kit (Waters, Milford, MA, USA). Amino acid analysis was performed using a Waters 2695 HPLC system (Waters, Milford, MA, USA) equipped with a 2998 PDA detector (Waters, Milford, MA, USA). The extraction of amino acids from the tea samples and the chromatographic conditions were the same as those described in our previous study [22].

#### 2.3.3. Determination of Non-Volatile Components

A mass of 50 mg of the sample was weighed into a 2 mL centrifuge tube, and 1200 μL of a 70% methanol aqueous solution pre-cooled to −20 °C was added. Every 30 min, the solution was vortexed, with each vortex lasting 30 s, for a total of 6 times. The solution was centrifuged at 12,000 rpm for 3 min, and the supernatant was aspirated to pass through a 0.22 µm filter membrane for UPLC-MS/MS analysis.

UPLC-MS/MS analyses were performed using a UPLC system (ExionLC^TM^AD, SCIEX, Shanghai, China) with a SB-C18 column (2.1 mm × 100 mm, 1.8 µm, Agilent Technologies, Santa Clara, CA, USA). Chromatographic conditions: column temperature 40 °C, flow rate 0.35 mL/min, injection volume 2 µL; mobile phase A was ultrapure water (containing 0.1% formic acid), mobile phase B was acetonitrile (containing 0.1% formic acid); the elution gradient was 0 min: 95% A, 5% B, 0–9 min: 5% A, 95% B, 9–10 min: 5% A, 95% B, 10–11 min: 95% A, 5% B, 11–14 min: 95% A, 5% B.

Mass spectrometry conditions: electrospray ionization (ESI) temperature 550 °C; ion spray voltage (IS) 5500 V (positive ion mode)/−4500 V (negative ion mode); ion source gas I (GSI), gas II (GSII), and curtain gas (CUR) were set at 50, 60, and 25 psi, respectively, and the collision-induced ionization parameter was set to high. QQQ scanning was performed using MRM mode with the collision gas (nitrogen) set to medium. In MRM mode, the first quadrupole selects the precursor ion (parent ion) of the target analyte, excluding ions of other molecular weights to eliminate initial interferences. The selected precursor ions then enter the collision cell, where they are induced to fragment into various product ions. These product ions subsequently pass through the third quadrupole, which filters and selects a specific characteristic fragment ion for detection, thereby excluding non-target ions. This two-stage filtration strategy significantly improves quantification, accuracy, and analytical repeatability. To monitor the repeatability of the entire analytical process, a quality control (QC) sample was inserted after every ten test samples.

#### 2.3.4. Data Analysis

All experiments were performed with three replicates. Bar graphs were generated using GraphPad Prism 5 software. Principal component analysis (PCA) and orthogonal partial least squares discriminant analysis (OPLS-DA) were performed using SIMCA 14.1 software. Heatmaps, Venn diagrams, radar charts, and correlation analyses were created using the online platform available at https://www.chiplot.online/.

## 3. Results and Discussion

### 3.1. Sensory Evaluation of Taste/Mouthfeel in Raw Pu-erh Tea Under Different Drying Methods

**Figure 1:**
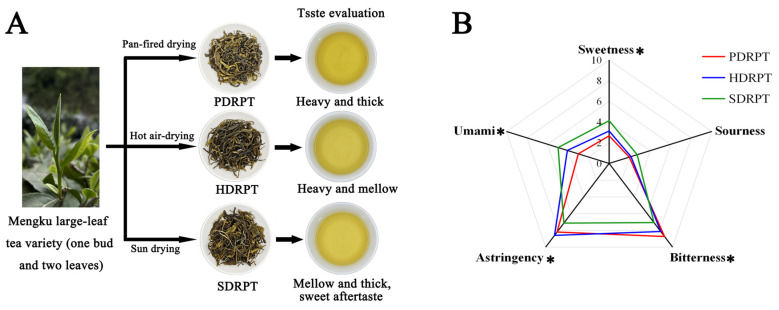
Sensory evaluation of taste/mouthfeel in raw Pu-erh tea under different drying methods. (**A**) Sensory description of taste/mouthfeel; (**B**) Radar chart of bitterness, umami, sweetness, sourness, and astringency intensities. “*” indicates significant differences (*p* < 0.05).

As shown in Figure 1A, raw Pu-erh teas under different drying methods exhibited considerable differences in taste/mouthfeel characteristics, while still maintaining the typical taste/mouthfeel characteristics of raw Pu-erh tea [23]. Specifically, PDRPT presented a heavy and thick mouthfeel, HDRPT presented a heavy and mellow mouthfeel, while SDRPT presented a mellow and thick mouthfeel with a sweet aftertaste. To further describe the taste/mouthfeel characteristics of the three raw Pu-erh teas in detail and accurately, the intensities of four basic taste attributes (bitterness, umami, sweetness, and sourness) and astringency were evaluated accordingly. As shown in Figure 1B, significant differences (*p* < 0.05) were observed in the intensities of bitterness, astringency, umami, and sweetness among the three raw Pu-erh teas under different drying methods. However, no significant difference was found in sourness. The bitterness and astringency intensities of PDRPT and HDRPT were significantly higher than those of SDRPT, while the umami and sweetness intensities of SDRPT were significantly higher than those of PDRPT and HDRPT.

### 3.2. Analysis of Main Biochemical Components in Raw Pu-erh Tea Under Different Drying Methods

#### 3.2.1. Bitter and Astringent Components

**Figure 2:**
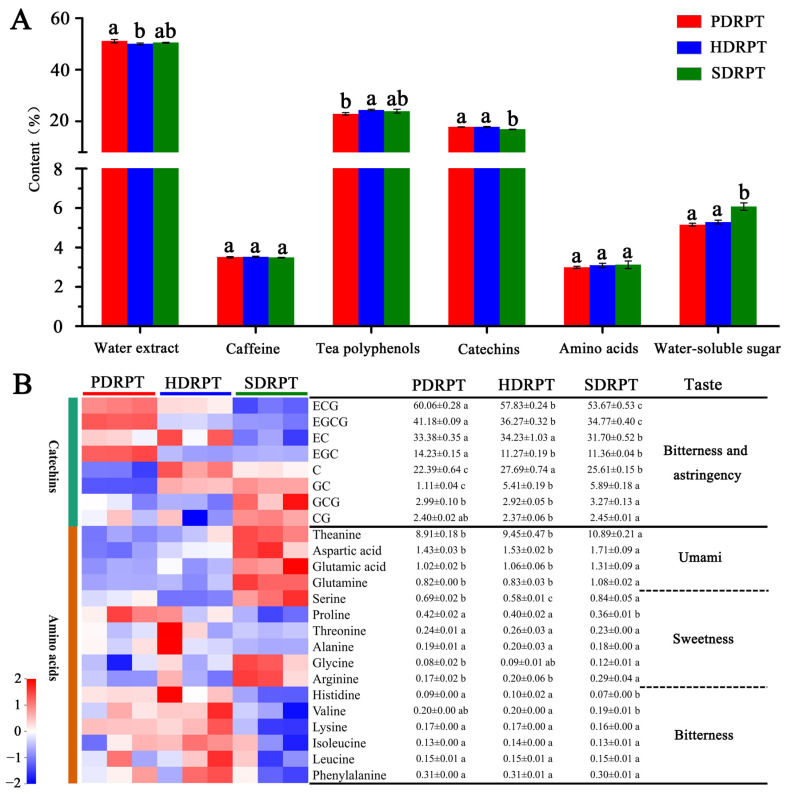
Analysis of main biochemical components in raw Pu-erh tea under different drying methods. (**A**) Contents of main conventional biochemical components (%); (**B**) Contents of catechins and amino acids (mg·g^−1^). Different lowercase letters indicate significant differences (*p* < 0.05).

Bitterness and astringency are the primary taste and mouthfeel characteristics of tea [24]. Generally, tea infusions with higher water extract content exhibit more pronounced bitterness and astringency, along with a fuller and richer taste/mouthfeel [25]. As shown in Figure 2A, PDRPT had the highest water extract content (51.14 ± 0.65%), while HDRPT had the lowest (50.03 ± 0.39%), though neither differed significantly from SDRPT. Caffeine and tea polyphenols are the main contributors to the bitterness and astringency of tea [6,24]. Figure 2A indicates that different drying methods had a certain impact on the tea polyphenols content of raw Pu-erh tea, but no significant effect on caffeine content. Specifically, HDRPT exhibited the highest tea polyphenol content (24.43 ± 0.23%), whereas PDRPT had the lowest (22.87 ± 0.47%), yet neither showed a significant difference compared to SDRPT.

Catechins are the main components of tea polyphenols, accounting for approximately 70% of the total tea polyphenol content. They are mainly characterized by bitterness and astringency in terms of sensory perception, and the intensity of these taste and mouthfeel attributes increases with higher concentrations [24,26]. As shown in Figure 2A, SDRPT had the lowest total catechins content at 16.87 ± 0.12%, which was significantly lower than that of PDRPT and HDRPT. Different types of catechins exhibit marked differences in the intensity of bitterness and astringency. For example, esterified catechins have strong bitterness, astringency, and convergence, whereas non-esterified catechins have a mellower mouthfeel and a refreshing aftertaste [24,26]. Figure 2B shows that the catechins of raw Pu-erh tea processed by the three drying methods generally exhibited the highest contents of ECG and EGCG, followed by EC, C, and EGC, while the contents of GCG, CG, and GC were relatively lower. In SDRPT, the contents of ECG, EGCG, and EC were significantly lower than those in PDRPT and HDRPT. This may be the key reason why the intensity of its bitterness and astringency is significantly lower than that of PDRPT and HDRPT. Previous research has also confirmed that the reduction in ECG and EGCG content is a crucial factor in the sun-drying process, reducing the bitterness and astringency of crude Ancha tea [9]. Concurrently, in SDRPT, the contents of the epicatechins (ECG, EGCG, EC, and EGC) were significantly lower than those in PDRPT, while the non-epicatechins (CG, C, GCG, and GC) showed an opposite trend. Similarly, compared with HDRPT, the epicatechins (ECG, EGCG, and EC) in SDRPT are also significantly decreased, whereas the non-epicatechins (CG, GCG, and GC) are significantly increased. This pattern is consistent with similar observations reported by Ye et al. in their study on the sun-drying process of An tea [9].

#### 3.2.2. Umami and Sweet Components

Umami and sweetness account for a relatively small proportion of the tea infusion’s taste profile, but they complement and balance the overall flavor, making them important factors in the formation of raw Pu-erh tea’s taste quality [24]. Amino acids are the main contributors to the umami taste of tea infusion and exhibit a certain correlation with its sweetness [6,27]. As shown in Figure 2A, the total amino acid content did not show significant differences among raw Pu-erh teas under different drying methods. However, tea is rich in various amino acids, and not all of them impart an umami taste; some amino acids contribute sweetness, while others even produce bitterness [6,27]. As shown in Figure 2B, a total of 16 amino acids were detected. Among these, theanine, aspartic acid, glutamic acid, glutamine, and serine were the predominant amino acids with relatively high contents, and their levels in SDRPT were significantly higher than those in PDRPT and HDRPT. Previous studies have suggested that the higher temperatures involved in pan-firing and hot-air drying promote the deamination, decarboxylation, and Maillard reactions of amino acids with carbonyl compounds, converting them into aromatic compounds such as pyrazines, thus leading to a decrease in amino acid content. In contrast, the relatively lower temperature of sun-drying is conducive to the retention of amino acids [10,28]. Among the five main amino acids mentioned above, except for serine, which contributes sweetness, the others are umami amino acids, and studies have confirmed their significant contribution to the umami taste of tea [27]. Therefore, the higher contents of theanine, glutamic acid, aspartic acid, and glutamine in SDRPT are the primary reasons for its more prominent umami characteristics.

Water-soluble sugars are the primary substances responsible for the formation of the sweet aftertaste in tea and can enhance the viscosity of the tea infusion [6,29]. Previous studies have shown that the total amount of monosaccharides decreases with increasing drying temperatures, suggesting that low-temperature drying methods, such as sun drying, are beneficial for the retention of monosaccharides [30]. During the high-temperature drying processes of hot-air drying and pan-fired drying, reducing sugars such as glucose participate in the Maillard reaction as reducing agents, contributing to the formation of the tea’s characteristic flavor [10]. As shown in Figure 2A, the water-soluble sugar content in SDRPT was 6.08 ± 0.19%, which was significantly higher than that in PDRPT and HDRPT, representing an increase of 17.84% and 14.87%, respectively. This result suggests that the higher content of water-soluble sugar is likely a significant reason for the more pronounced sweet aftertaste observed in SDRPT.

### 3.3. Analysis of Non-Volatile Components Profile in Raw Pu-erh Tea Under Different Drying Methods

**Figure 3:**
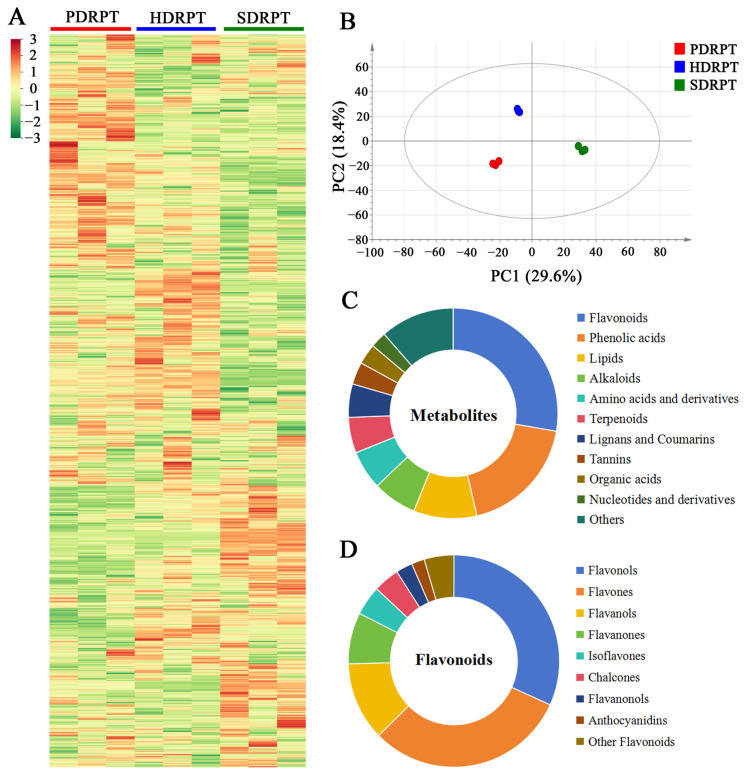
Analysis of non-volatile components in raw Pu-erh tea under different drying methods; (**A**) Heat map of the relative content of non-volatile components; (**B**) PCA score plot derived from the relative content of non-volatile components; (**C**) Composition of non-volatile components; (**D**) Composition of flavonoid components.

As shown in Figure 3A, a total of 1987 non-volatile components were identified in raw Pu-erh tea under three different drying methods, and these components exhibited considerable differences in their relative contents. PCA showed that the three groups of tea samples, PDRPT, HDRPT, and SDRPT, were clearly separated on the principal component score plot (Figure 3B), indicating that different drying methods have a significant impact on the non-volatile components of raw Pu-erh tea. In terms of non-volatile component categories (Figure 3C), the 1987 non-volatile components mainly included 551 flavonoids, 370 phenolic acids, 194 lipids, 135 alkaloids, 119 amino acids and derivatives, 109 terpenoids, 102 lignans and coumarins, 68 tannins, 63 organic acids, 49 nucleotides and derivatives, and 227 others. Among these, flavonoids were the most abundant components, mainly consisting of 175 flavonols, 170 flavones, 66 flavanols, 43 flavanones, 25 isoflavones, 22 chalcones, 14 flavanonols, 11 anthocyanidins, and 25 other flavonoids (Figure 3D).

### 3.4. Analysis of Differential Non-Volatile Components in Raw Pu-erh Tea Under Different Drying Methods

**Figure 4:**
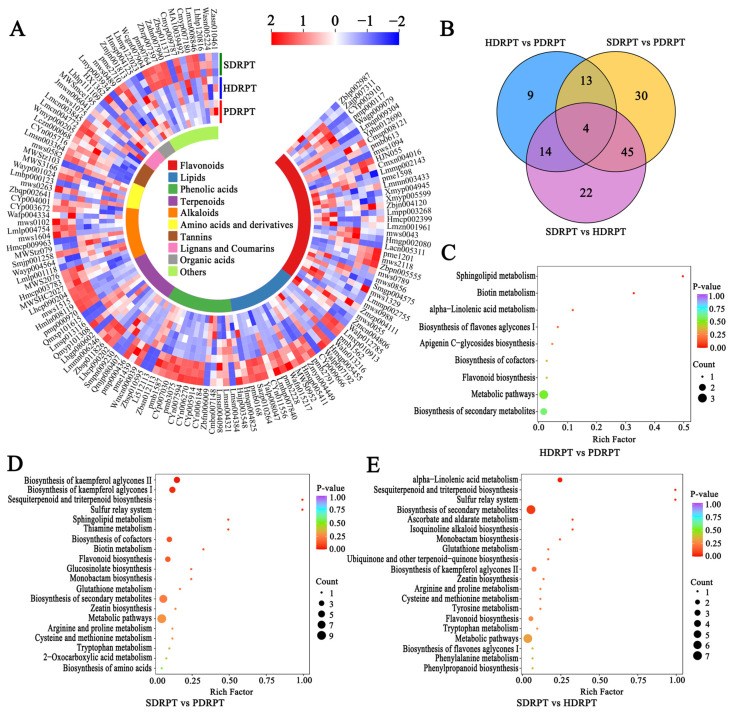
Analysis of differential non-volatile components in raw Pu-erh tea under different drying methods. (**A**) Heat map of the relative contents of differential non-volatile components; (**B**) Venn diagram of the number of differential non-volatile components in the comparisons between HDRPT and PDRPT, SDRPT and PDRPT, SDRPT, and HDRPT; (**C**) KEGG enrichment of differential metabolites between HDRPT and PDRPT; (**D**) KEGG enrichment of differential metabolites between SDRPT and PDRPT; (**E**) KEGG enrichment of differential metabolites between SDRPT and HDRPT.

OPLS-DA was applied to distinguish and analyze the differential non-volatile components among PDRPT, HDRPT, and SDRPT. The screening of differential non-volatile components was based on a variable importance in the project (VIP) ≥ 1, a fold change ≥ 2 or fold change ≤ 0.5, and *p* < 0.05. As shown in Figure 4A, a total of 137 differential non-volatile components were identified, including 36 flavonoids, 19 lipids, 18 phenolic acids, 14 terpenoids, 14 alkaloids, 7 amino acids and derivatives, 6 tannins, 5 lignans and coumarins, 4 organic acids, and 13 others. The relative contents of the vast majority of these differential non-volatile components, particularly lipids, phenolic acids, and terpenoids, were significantly higher in SDRPT compared to PDRPT and HDRPT. Compared to SDRPT, the higher drying temperatures used for PDRPT and HDRPT led to substantial degradation and loss of heat-unstable lipids, phenolic acids, and terpenoids, whereas SDRPT was able to better retain these components. Previous studies have confirmed that lipids, phenolic acids, and terpenoids participate together in the overall construction of tea infusion taste through multiple pathways, such as direct flavor contribution and indirect modification, making them indispensable components of tea taste quality [6,31,32]. Based on this, it is speculated that the relatively enriched lipids, phenolic acids, and terpenoids in SDRPT may constitute an important material basis for the formation of its unique taste quality.

Venn diagram analysis results (Figure 4B) showed that the numbers of differential non-volatile components in the HDRPT vs. PDRPT, SDRPT vs. PDRPT, and SDRPT vs. HDRPT groups were 40, 92, and 85, respectively, and the numbers of unique differential components were 9, 30, and 22, respectively. This indicates that different drying methods have varying degrees of impact on the non-volatile components of raw Pu-erh tea, with the degree of difference between HDRPT and PDRPT being significantly lower than that between either of them and SDRPT. Further pathway enrichment analysis of the differential non-volatile components (Figure 4C–E) revealed that the HDRPT vs. PDRPT, SDRPT vs. PDRPT, and SDRPT vs. HDRPT groups were enriched in 9, 21, and 24 pathways, respectively. The number of enriched pathways in HDRPT vs. PDRPT was significantly lower than that in SDRPT vs. PDRPT and SDRPT vs. HDRPT, which was consistent with the results of the differential component analysis. Notably, the SDRPT vs. PDRPT and SDRPT vs. HDRPT groups exhibited high consistency in their enriched pathways, sharing 15 common pathways, mainly including biosynthesis of secondary metabolites and glutathione metabolism.

### 3.5. Correlation Analysis of Differential Non-Volatile Components with Main Taste/Mouthfeel Attributes

**Figure 5:**
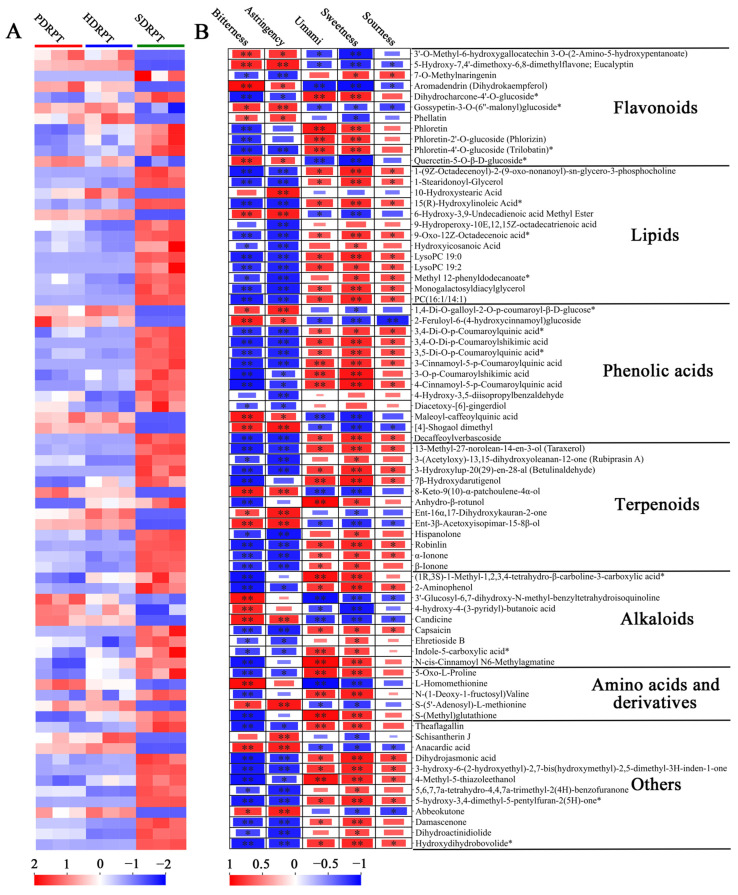
Correlation analysis of differential non-volatile components with main taste/mouthfeel attributes. (**A**) Relative contents of differential non-volatile components; (**B**) Heatmap of correlation coefficients. “*” indicates significant differences (*p* < 0.05), “**” indicates highly significant differences (*p* < 0.01).

As shown in Figure 5A, a total of 75 non-volatile components were screened in SDRPT that exhibited significant differences compared to both PDRPT and HDRPT. These included 11 flavonoids, 13 lipids, 13 phenolic acids, 12 terpenoids, 9 alkaloids, 5 amino acids and derivatives, and 12 others. The relative contents of the vast majority of these differential components were higher in SDRPT than in PDRPT and HDRPT, with this trend being particularly prominent for lipids, phenolic acids, and terpenoids. We speculated that this may be because SDRPT is dried at a relatively low temperature, which allows for better retention of the biochemical components in the tea leaves, particularly the heat-labile lipids, phenolic acids, and terpenoids. To further understand the key material basis for the formation of the unique taste/mouthfeel quality of SDRPT, a correlation analysis was conducted between the relative contents of these 75 differential non-volatile components and the intensities of bitterness, astringency, umami, sweetness, and sourness. As shown in Figure 5B, all 75 differential non-volatile components exhibited a significant positive or negative correlation with at least one of these attributes. Most of these differential components showed a significant positive correlation with umami, sweetness, and sourness attributes, while displaying a significant negative correlation with bitterness and astringency attributes.

Notably, some of these differential components have been confirmed to contribute to the formation of tea taste/mouthfeel quality. Among the eleven differential flavonoids screened, five showed a significant positive correlation with umami and sweetness. Although flavonoids are considered the main source of bitterness and astringency in tea infusions [24], dihydrochalcones such as phloretin, phloretin-2′-O-glucoside, phloretin-4′-O-glucoside, and dihydrochalcone-4′-O-glucoside possess natural high-intensity sweetness properties and are key substances responsible for the formation of the sweet aftertaste and mellow mouthfeel in *Camellia nanchuanica* black tea and sweet tea [33,34]. Among the 13 differential lipids screened, up to 11 showed a significant negative correlation with bitterness and astringency. Nakayama et al. [35] indicated that catechins can specifically interact with the choline groups of phospholipids through their galloyl moieties, thereby attenuating the perception of bitterness and astringency in tea infusions. Based on this, it is speculated that differential lipid components such as LysoPC 19:0 and LysoPC 19:2 may bind to bitter and astringent catechins through a similar mechanism, reducing their free concentration in the tea infusion and thus inhibiting bitterness and astringency. Among the thirteen differential phenolic acids screened, nine showed a significant negative correlation with bitterness and astringency. Ye et al. [9] similarly found that all differential phenolic acids in three types of crude Ancha tea (hot-air dried, sun-dried, and combination-dried) were significantly negatively correlated with bitterness and astringency. Although phenolic acids typically exhibit a sour and astringent, their content is positively correlated with the degree of tea oxidation, a process that reduces bitterness and astringency; moreover, an appropriate amount of phenolic acids and other organic acids can enhance the mellow mouthfeel of tea infusions [6,36]. Among the twelve differential terpenoids screened, up to nine showed a significant positive correlation with umami and sweetness. Although terpenoids primarily influence tea quality through their aroma contribution, their effect on enhancing tea infusion taste/mouthfeel has been increasingly reported recently [32,37,38]. For example, Wei et al. [38] identified that aroma components such as (E)-β-damascenone, linalool, dihydroactinidiolide, and (E)-β-ionone could enhance the sweetness of tea infusions, increasing the sweetness by over 24.0%. In summary, the 75 differential non-volatile components collectively participate in the formation of the unique taste/mouthfeel of SDRPT through multiple pathways, including direct flavor contribution and indirect modification.

## 4. Conclusions

In this study, the drying methods significantly altered the sensory taste/mouthfeel characteristics of raw Pu-erh tea. PDRPT exhibited a heavy and thick mouthfeel, while HDRPT presented a heavy and mellow mouthfeel, with both showing significantly higher bitterness and astringency intensities than SDRPT. In contrast, SDRPT displayed a mellow and thick mouthfeel with a sweet aftertaste, and significantly higher umami and sweetness intensities compared to PDRPT and HDRPT. The key material basis for the unique taste/mouthfeel quality of SDRPT includes relatively higher contents of amino acids such as theanine, glutamic acid, aspartic acid, and glutamine, as well as water-soluble sugars, combined with relatively lower contents of catechins such as ECG and EGCG. Furthermore, a total of 1987 non-volatile components were identified, and 137 differential metabolites were screened. Among these, 75 components showed significant differences in SDRPT compared to both PDRPT and HDRPT. The relative contents of the vast majority of these differential components were higher in SDRPT than in PDRPT and HDRPT, with this trend being particularly prominent for lipids, phenolic acids, and terpenoids. Correlation analysis revealed that all 75 differential components were significantly positively or negatively correlated with at least one taste/mouthfeel attribute. Most of these differential components showed a significant positive correlation with umami, sweetness, and sourness attributes, while displaying a significant negative correlation with bitterness and astringency attributes. These findings provide a theoretical basis for elucidating the formation mechanism by which drying processes influence the taste/mouthfeel quality of raw Pu-erh tea and also offer a scientific foundation for improving its quality. Notably, this study has limitations, including limited sensory data coverage and insufficient mechanistic depth (e.g., the aroma-taste linkage remains unclear). These limitations nonetheless point to clear future research directions. We will address these issues and further explore the relevant topics in subsequent studies.

## Data Availability

The original contributions presented in the study are included in the article. Further inquiries can be directed to the corresponding authors.
